# Easy-to-Read: Evolution and Perspectives—A Bibliometric Analysis of Research, 1978–2021

**DOI:** 10.3390/ijerph20043359

**Published:** 2023-02-14

**Authors:** Marcela Alina Fărcașiu, Vasile Gherheș, Simona Șimon, Daniel Dejica-Carțiș, Liviu Cădariu, Annamaria Kilyeni

**Affiliations:** 1 Department of Communication and Foreign Languages, Politehnica University of Timisoara, 300006 Timisoara, Romania; 2 Mathematics Department, Politehnica University of Timisoara, 300006 Timisoara, Romania

**Keywords:** easy-to-read, easy read, intellectual (learning) disabilities, accessibility, social exclusion, inequities, bibliometric analysis

## Abstract

The purpose of this paper is to observe the use of the Easy-to-Read term in the international scientific literature. Therefore, a bibliometric analysis was carried out using the Web of Science database, focusing on the period between 1978 and 2021. From this, 1065 records that met the search criteria were further identified. After applying the PRISMA model, the final analysis was performed on a corpus of 102 documents, comprising an analysis of the keywords and expressions where the term was found, an authorship analysis, a citation analysis, as well as a co-occurrence analysis. The publications were grouped based on the research area, with the field of Computer Science standing out with most of the occurrences (25), followed by Education & Educational Research (14 occurrences) and Linguistics (9 occurrences). The results suggest that interest in this field of research is limited, as the maximum number of publications related to this topic was 16 in 2020 and 14 in 2021. The study is important as it sheds light on the current state of the topic and seeks to identify future trends in this field.

## 1. Introduction

The United Nations (UN) Convention on the Rights of Persons with Disabilities (UNCRPD) [1], adopted in December 2006, was the first tool created to help people with disabilities, i.e., ‘those who have long-term physical, mental, intellectual or sensory impairments which in interaction with various barriers may hinder their full and effective participation in society on an equal basis with others’ (Article 1). It stipulates, among other things, that these people must be included in the community (Article 19), must have equal access to education and training (Article 24), and that they must have access to transportation, public facilities, and services, as well as information and communication technologies (Article 9). The EU also promotes these rights and the inclusion and participation of people with disabilities in society through the Charter of Fundamental Rights of the EU [2] and, in March 2021, the European Commission adopted a new Strategy for the Rights of Persons with Disabilities 2021–2030 [3], which builds on the European Disability Strategy 2010–2020 [4].

According to the latest statistics, 87 million Europeans, i.e., one in four European adults, have a disability, with Malta having the lowest prevalence (11%) and Latvia the highest prevalence (39.5%) of disabled people. These rates are also reflected in the higher unemployment rates, poverty and social exclusion, lack of education, and discrimination experienced by disabled people [5].

The new Strategy for the Rights of Persons with Disabilities 2021–2030 establishes an Action Plan based on web accessibility with the purpose of creating compliance across all the EU websites, the documents published on these websites and online platforms, and publications in an Easy-to-Read format [3]. Moreover, the Web Accessibility Directive [6] has been enforced since 2016, requiring websites and public sector institutions to comply with specific accessibility standards in order to ensure social inclusion for all. Member States had until the end of 2018 to implement the Directive into their own countries.

**Figure 1:**
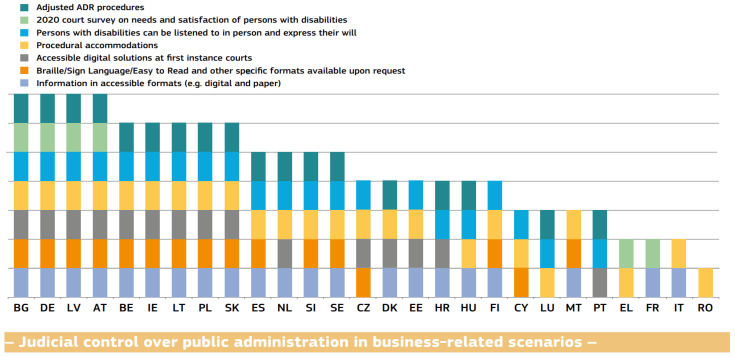
Specific arrangements for access to justice of people with disabilities in the EU [7].

If we take justice as an example, the 2022 EU Justice Scoreboard shows how justice can be accessed by people with disabilities in different Member States. As can be seen in the figure below (Figure 1), information is available in Braille, sign language, or Easy-to-Read language, as well as in other accessible formats (digital and paper) in more than half of the Member States.

People with intellectual disabilities are a specific group that experience lower rates of employment, with only 50.8% of the demographic being employed, as opposed to 74.8% of people without disabilities [8]. These people face the risk of social exclusion, which could be prevented, at least partly, by facilitating their access to information through documents created in a simplified and understandable manner both from a linguistic and a technical point of view, i.e., in an Easy-to-Read and Easy-to-Understand manner.

For example, in Romania, a member of the European Union, in 2020, out of 1544 public institutions (social services), only 3 of 1441 institutions were accessible from an informational point of view, and only 76 of 1306 institutions (5%) were accessible as far as communication is concerned. At the same time, only 12% of these institutions used certified interpreters for sign language or interpreters for people with deaf-blindness, and only in 32% of cases was there a person designated to assist people with disabilities; moreover, even though the National Authority for the Rights of People with Disabilities, Children and Adoptions (ANDPDCA) has requested the implementation of a guidebook on writing all public documents in an Easy-to-Read and Easy-to-Understand format, this has not yet been accomplished [9,10,11]. This is reflected in Figure 1, wherein Romania ranks last in making justice accessible to people with disabilities.

Providing accessible documents for people with disabilities is, thus, an obligation that many Member States are still yet to fulfill. Access to information is a right of all citizens, and, for this to be achieved, information needs to be in an inclusive format which allows people with disabilities to live independently and to participate actively in the community. The lack of accessibility is a major problem that generates the discrimination and isolation of people with disabilities. Policies and practices that ensure equal opportunities and chances for socially excluded or marginalized people to improve their quality of life and to increase their employment rate are, therefore, needed. 

Concerns regarding the accessibility of information for people with intellectual disabilities are relatively recent: the concept encompassing it, *Easy-to-Read*, came to the attention of researchers only in the first decade of this century. The term has given rise to different interpretations, as there is no widespread use and no unitary understanding around it. Therefore, the research team has decided to answer the question: “What are the trends and the evolution of the research on the Easy-to-Read term throughout the time?” in order to provide information that can help improve research strategies in this field. 

This paper seeks to capture the use of the Easy-to-Read term in the international literature (journals, proceedings papers and other documents related to academic disciplines), or, more precisely, in the ISI Web of Science database. Starting from the definition of the concept, this research then focuses on a bibliometric analysis of the literature in this field in order to see both the importance of the term and the type of research tackling it with the purpose of making documents and websites more accessible for a better integration of the people with disabilities into their society.

## 2. Easy-to-Read: Definition and Terminology

The Easy-to-Read language, whose beginnings date back to the 1970s’ ‘People First’ movement for the rights of the people with disabilities, encompasses a set of rules that help simplify the text in order to make it easier to read and understand [12]. Besides people with intellectual disabilities (e.g., autism) and learning disabilities (e.g., dyslexia), who are the main beneficiaries of these texts, other categories of people might be the recipients of such simplified texts (e.g., the elderly, recent immigrants or migrants, or low-literate people) [12,13,14]. 

*Easy-to-Read*, *Easy Read*, *Easy Write*, *Easy Info*, *Easy Access*, *Aphasia friendly* [15] are all terms used to refer to simplified language in a specific format employed for the above-mentioned categories of people that have difficulty reading or understanding information due to certain intrinsic or extrinsic factors. In the UK, Australia, and New Zealand, *easy read* is preferred instead of Easy-to-Read [16]. Easy-to-Read has specific terms in different languages (*Lättläst* in Sweden, *Selkokieli* in Finnish, *Lectura Fácil* in Spain, *Linguaggio facile da leggere* in Italy, *Leichter Sprache* in Germany, etc.), as the concept has been implemented in the Scandinavian countries since the 1970s and thus is more developed compared to other European countries [17,18,19,20,21].

In opposition, *plain language, plain writing*, *layman terms*, *layperson terms* [15] are terms meant to describe information that is presented in a simpler way (e.g., legal and governmental documents) for people with literacy skills, who can read and write reasonably, but who are not familiar with some jargon and complex terms.

Despite all the above, some scholars use Easy-to-Read in a general way, meaning “more legible”, “simpler”, “more intelligible”, “more coherent”, with its use overlapping with that of plain language. There are, for example, academic papers using this term in contexts that are not related to the categories of people discussed in the Section 1 [22,23].

Another use of the Easy-to-Read language with a different meaning is its abbreviated form (E2R)—also used in the field of accessibility—used in medicine and meaning “Estradiol receptor” [24,25]. 

## 3. Methodology

The publications (e.g., articles, proceeding papers, review articles, book chapters, early access articles, editorial materials) that approached the topic of Easy-to-Read were searched in the Web of Science (WoS) database. The search was conducted in the Web of Science database because this includes more than 21,000 academic journals in more than 250 disciplines such as sciences, social sciences, and arts and humanities [26]. Since, in the scientific literature, the term Easy-to-Read is written in different forms, in the Web of Science Core Collection, the search was carried out in titles, abstracts, and keywords using the following expressions: “easy-to-read”, “easy to read”, “easy read”, and “E2R”. The data were collected in mid-March 2022 and included all the information recorded from the beginning of 1978 until the end of 2021 (the information for the year 2022 was not collected because the year was not yet over). The VOSviewer tool (version 1.6.10) was used to carry out different analyses regarding research areas, co-words, authorship, authors’ main affiliation, citations, keywords, and their co-occurrences.

## 4. Data Analysis and Discussion

**Figure 2:**
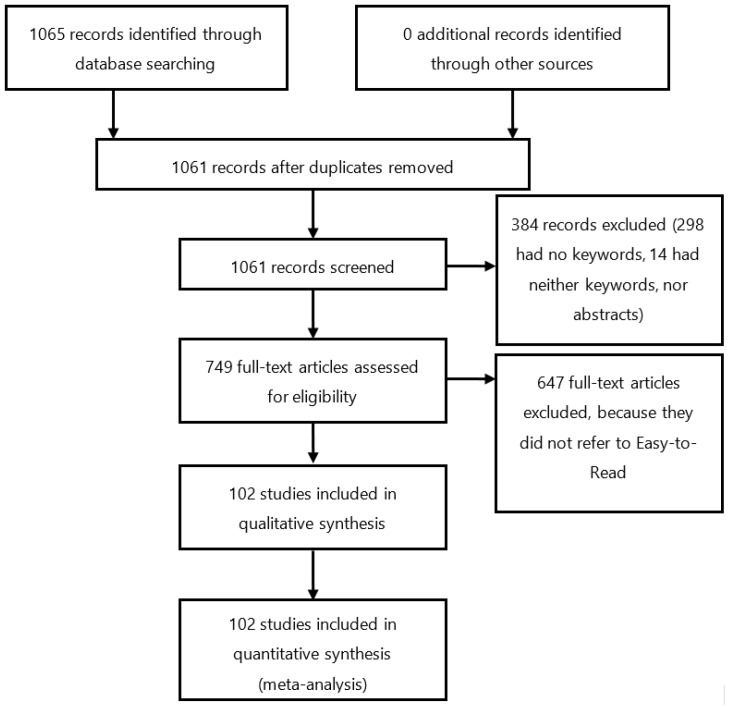
PRISMA guidelines describing the collection of documents from Web of Science.

After having applied the PRISMA model [27], 102 works were identified (Figure 2). From 1065 records that met the search criteria for which the metadata records were downloaded, 4 duplicates, 298 articles without any keywords, and 14 with neither keywords nor abstracts were removed. Following the analysis of the abstracts, from the total of 749 records, 647 records were excluded because they were unrelated to the concept of Easy-to-Read (see the Section 1 above). 

### 4.1. Annual Trends of Easy-to-Read-Related Publications

**Figure 3:**
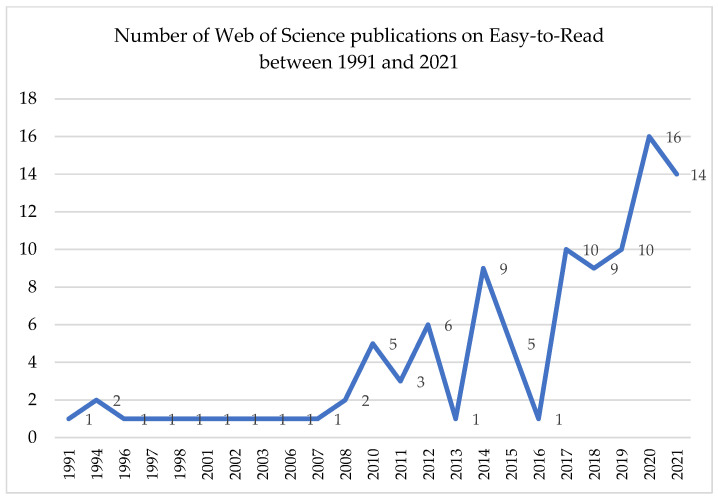
Number of Web of Science publications on Easy-to-Read between 1991 and 2021.

Figure 3 below shows the annual trends in the number of publications published between 1978 and 31 December 2021. As can be seen, the interest in this field was quite limited before 2010, with the maximum number of 16 publications being reached in 2020. Moreover, 2021 had a similarly high number of publications (14 publications). Throughout the studied interval, the topic of Easy-to-Read has received greater attention from the academic community in the last 10 years (namely, since 2012).

### 4.2. Research Areas

An analysis was also carried out on the research area, with the figures in the graph representing the number of occurrences in that field. In the *Other* category, the fields in which a single occurrence was recorded were included as follows: Arts (1), Endocrinology & Metabolism (1); General & Internal Medicine (1); Genetics & Heredity (1); Health Care Sciences & Services; Biomedical Social Sciences (1), Geriatrics & Gerontology; Pharmacology & Pharmacy (1), Infectious Diseases (1), Social Sciences—Other Topics (1).

**Figure 4:**
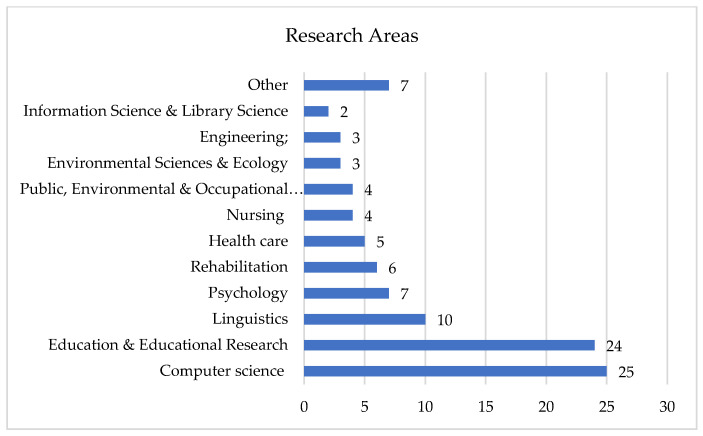
Distribution of publications based on research area.

As Figure 4 shows, the field in which the most publications are found is Computer Science (19 occurrences), to which, in order to simplify the graphic representation, the following fields have been added: Computer Science Education & Educational Research; Rehabilitation (one occurrence), Computer Science; Engineering (four occurrences), and Computer Science; Engineering; Telecommunications (one occurrence). The main field, Computer Science, cumulated all the above results, with the total of occurrences being 25. 

**Figure 5:**
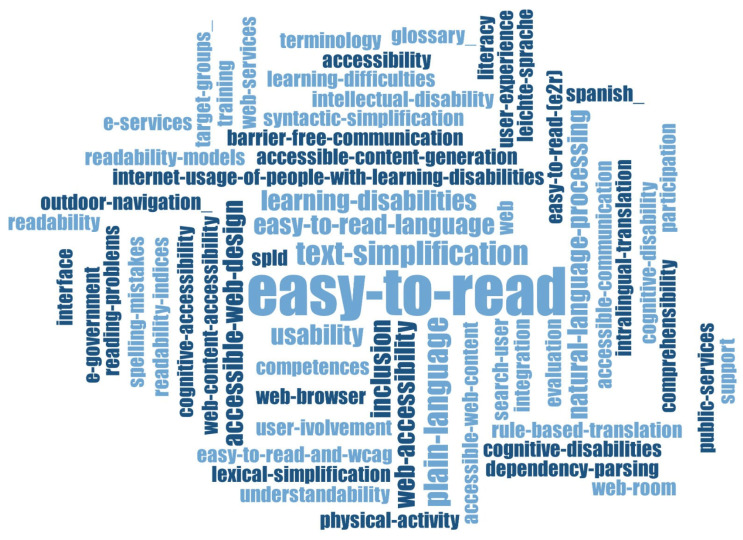
Word cloud generated from publication keywords of selected publications from Computer Science area based on the frequency of each keyword.

The terms usually associated with this field of research, as presented in Figure 5 below, are related to the web category (*web accessibility, web content, web browser, interface*), to its accessibility (*accessible web content, accessible web design, accessible content generation*), to the type of users it is designed for (having *cognitive disabilities, intellectual disabilities, learning difficulties*), to the type of language needed for these categories of people (*easy-to-read language*), and to the purpose of this research (*text simplification, readability, understandability, user involvement, inclusion, integration*).

Most of the publications in this category demonstrate an awareness of the fact that people with various cognitive disabilities need to be able to use websites that have to be accessible to them from a linguistic and technical point of view (larger fonts, simplified layout, highlighted text) [28,29,30,31,32].

The most cited study [33] of the researched database presents the guidelines created by the W3C Web Accessibility Initiative with the purpose of making websites more accessible from a readability point of view by using the Easy-to-Read text guidelines for people with various disabilities.

The importance of using the Easy-to-Read language, and therefore the establishing of guidelines for web accessibility for people with cognitive and learning disabilities, is also emphasized by Schmutz et al. [34]. The use of the Easy-to-Read format to help with website accessibility is also advocated for e-government systems, which are mandatory for the citizens’ relationship with the authorities [35]. 

The next most frequent category was Education & Educational Research (14 occurrences). In this category, other fields were added, such as Education & Educational Research; Genetics & Heredity; Neurosciences & Neurology; Psychiatry; Rehabilitation (1 occurrence), Education & Educational Research; Mathematics (1 occurrence), Education & Educational Research; Psychology (2 occurrences) and Education & Educational Research; Rehabilitation (6 occurrences). 

**Figure 6:**
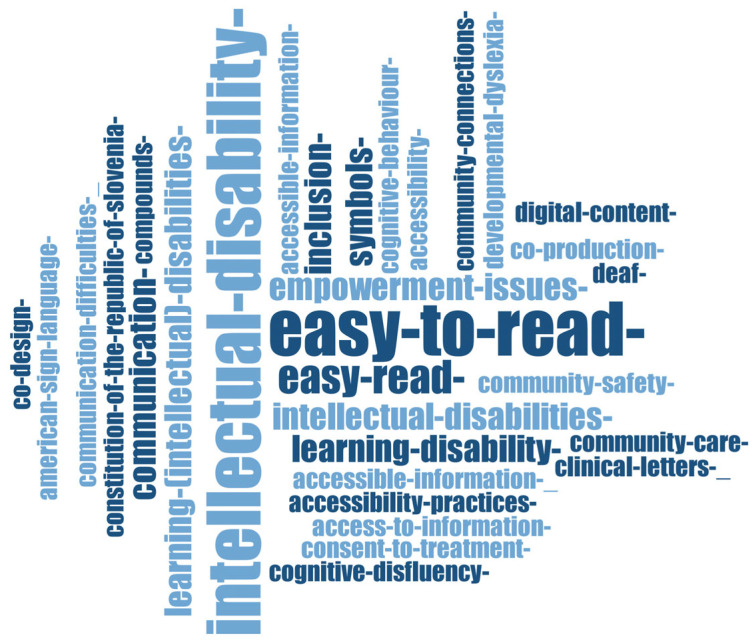
Word cloud generated from publication keywords of selected publications from Education & Educational Research area based on the frequency of each keyword.

The terms that scholars make use of in this research area (Figure 6) refer to the accessibility (*accessible communication, accessible information*) of resources for different types of people (*immigrant children*, those with *functional illiteracy*, having *learning (intellectual) disabilities* or *cognitive disabilities*, with *dyslexia*), through *Easy-to-Read* (*lexical simplification*, *semantic transparency*, *symbols*, *syntactic simplification*, *visual support*) for *barrier-free communication*, *community care, empowerment*, *inclusion*, *independent living*, *integration*, *user involvement*, and against *social exclusion*.

This category of research focuses on conveying that the Easy-to-Read language is mandatory for readability literacy, especially for immigrant children [36] and people with learning (intellectual) disabilities [37,38]. The problem of accessibility is also raised here, with people with a learning disability being faced with social exclusion [39]. Teaching students with special educational needs should be performed with the use of the Easy-to-Read language and visuals in order to include them in the educational process [40,41]. The same applies for the study on people with dyslexia [42]. 

A part of the research in this field relates to Health Education. For people with intellectual disabilities, accessible health materials must be created in order to facilitate their comprehension of the medical system and processes [43,44,45].

The field of Linguistics ranks third (9 occurrences), to which Linguistics; Literature is also added (1 occurrence), with this category totaling 10 occurrences.

**Figure 7:**
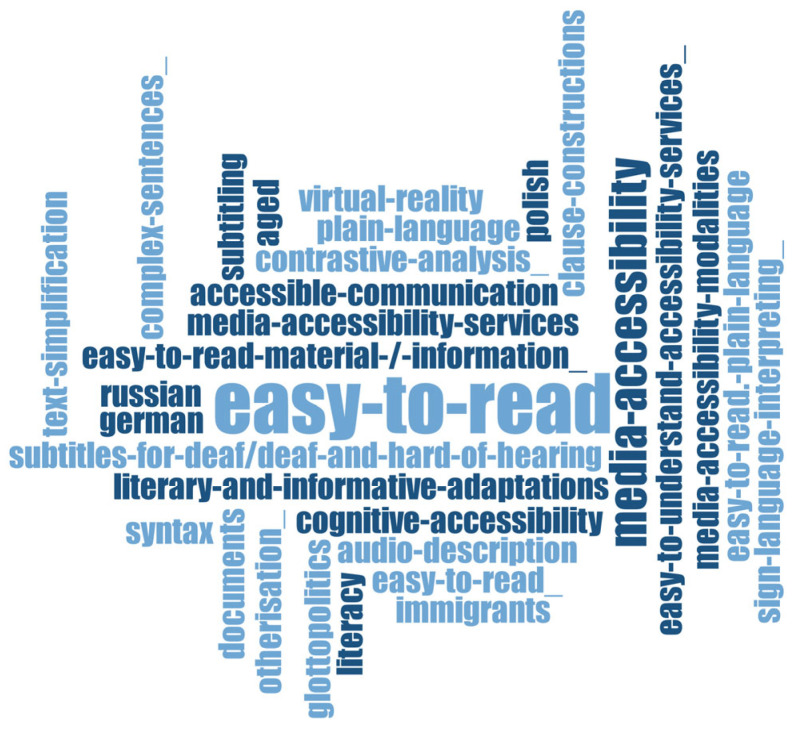
Word cloud generated from publication keywords of selected publications from Linguistics Research area based on the frequency of each keyword.

The terms used by scholars in this field (Figure 7) refer to the Easy-to-Read term discussed in various languages (*German, Polish, Russian, Spanish*) encompassing everything related to its use (*accessible communication*, *cognitive accessibility*, *media accessibility services*, *audio description*, *subtitles for deaf/deaf*, and *hard-of-hearing*).

This field is organized around the explanations and description of the concept of Easy-to-Read and its use in different types of materials in different countries in Europe, e.g., in Germany [46], in Russia [47], and in Spain [48,49]. The scholars’ interest has also been focused on audiovisual linguistic accessibility, e.g., audio description [50], media accessibility services [51], or subtitling for deaf and hard-of-hearing people [52].

### 4.3. Authorship Analysis

**Figure 8:**
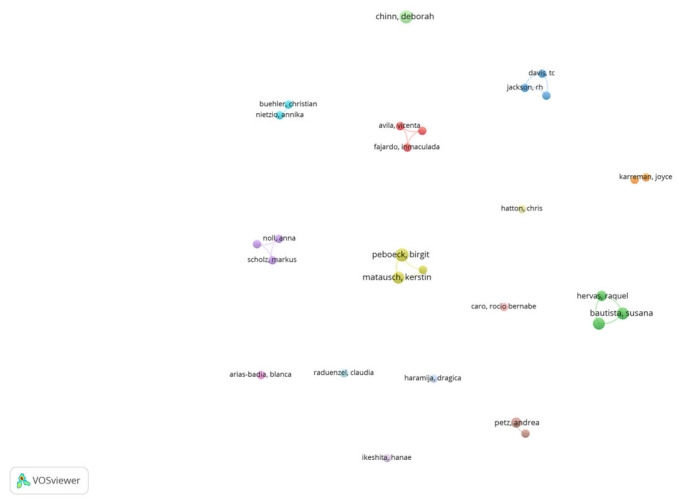
Map of authors authoring more than two publications.

The publications were also analyzed to obtain information about their authors, and the result of this was that the 102 publications had a total of 315 authors. The VOSviewer tool was used to analyze and map the information. Figure 8 shows the authors who authored at least two publications; two authors authored five publications each (Chinn and Peboeck), three authors authored four publications each (Bautista, Gervas, and Matausch), and two authors authored three publications each (Hervas and Petz). A further 21 authors authored two publications each, and 287 authors authored only one publication. 

### 4.4. Analysis of the Main Affiliation of the Authors

**Figure 9:**
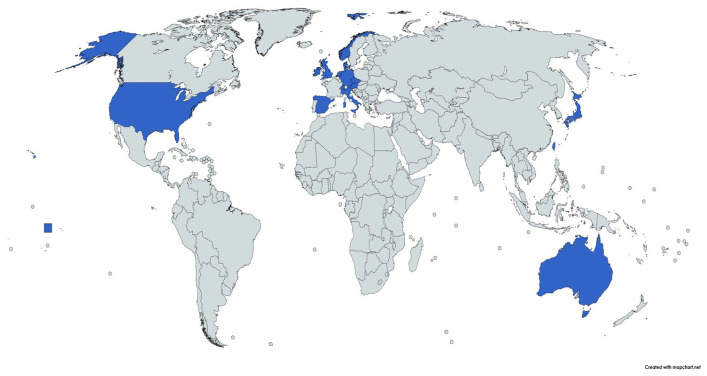
Map of authors’ main affiliation.

Based on the country of origin of the first author of the publications, 24 countries were singled out, with the geographical distribution being created only for the countries that had at least two documents (in blue, see Figure 9). The most prolific country was Great Britain with 24 publications, followed by Germany (21), Spain and the USA (14), Austria (9), Australia (5), and the Netherlands (4). Ireland, Slovenia, and Taiwan accounted for only 3 publications each, and 2 publications were recorded in the case of Denmark, Italy, Japan, Norway, and Switzerland.

### 4.5. Citation Analysis

**Figure 10:**
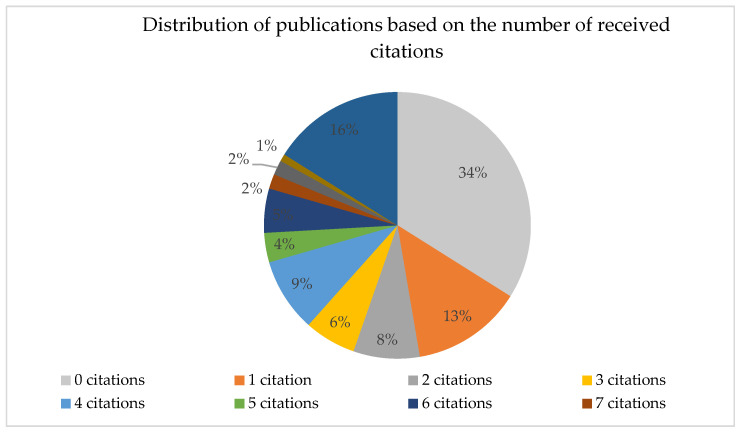
Distribution of publications based on the number of received citations.

The 102 selected publications received 858 citations in total, with an average of 11.59 citations per publication. The highest share is represented by the category of publications that did not receive any citations (34%), followed by those that exceeded 10 citations each (16%) and those that received only one citation (13%). The distribution of all citations can be seen in Figure 10.

The number of received citations in publications by country places the USA first with 244 citations and Great Britain second with 197 citations, these being followed in a descending order by Spain (49), Australia (47), the Netherlands (43). 

The publications exceeding an average value of 11.59 citations per publication were also highlighted (Table 1 below). As can be observed, the highest number of citations is found in publications pertaining to the Health Care research area, dealing with the topic of information accessibility for people with low literacy. 

### 4.6. Keyword Analysis

Keywords provided by the authors of the publications and appearing more than five times in the Web of Science database were included in the final analysis. Of the 473 keywords, 11 reached the threshold mentioned above. The keywords that appeared alongside the concept of Easy-to-Read, in all its forms, with the frequency of occurrence in brackets, are the following: *easy-to-read (19), intellectual disability (15), accessibility (9), easy-to-read language (8), plain language (8), easy read (7), intellectual disabilities (6), readability (6), text simplification (6), easy to read (5),* and *natural language processing (5)*.

The concept was also found in the keywords in the form of an expression, the frequency of appearance being included in brackets: *easy-to-read language (8), easy-to-understand language (2), easy to read guide (1), easy-to-read and wcag (1), easy-to-read material/information (1), easy-to-read materials (1), easy-to-read text (1), easy-to-read texts (1), easy-to-understand accessibility services (1).*

### 4.7. Co-Occurrence Analysis

**Figure 11:**
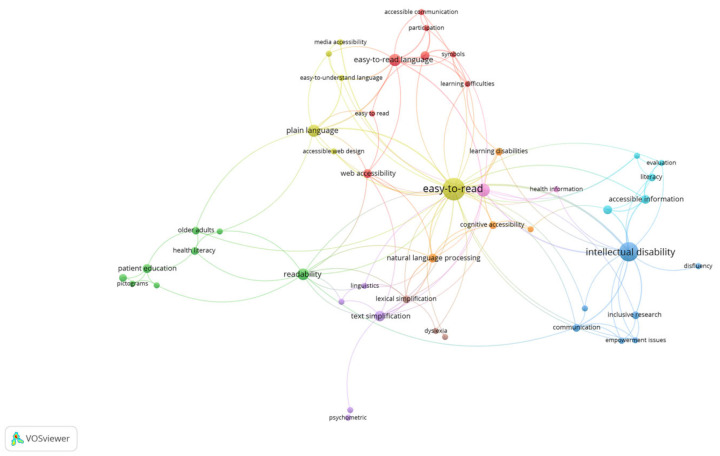
Co-occurrence of keywords in the researched publications.

A keyword co-occurrence analysis (Figure 11) was also carried out using VOSviewer to show the topics covered by the publications, with emphasis on the ones that received more attention from the authors, suggesting possible future research directions. The analysis was undertaken by applying two minimum occurrences of a word; 50 keywords were found, which were then classified into nine clusters. The most used words were “Easy-to-Read” (26 times) and “intellectual disability” (19 times). 

The red cluster gathers publications dealing with the main purpose of the Easy-to-Read language, i.e., inclusion of people with different types of disabilities, and touches on the importance of creating such documents for people with low literacy skills and for people with cognitive disabilities for web accessibility purposes. The green cluster provides a very important link between the readability of health materials for low literate and older people and patient education, aiming for their integration into a normal life, emphasizing once again the importance of the Easy-to-Read language.

The blue cluster’s research centers around people with intellectual disabilities (mainly learning disabilities) and the way in which materials (both video and print ones) are communicated to them (in an Easy-to-Read format) in order to empower them to be able to function in all of society’s fields of activity as well as on inclusive research that is performed with the purpose of integrating them and preventing their social exclusion. 

Easy-to-Read, being the main keyword in the yellow cluster, reinforces the message that media accessibility, with everything that it entails, e.g., accessible web design or audio description, is paramount for the creation of an inclusive society. The Spanish scholars’ linguistic efforts to devise a text simplification method to help people with learning disabilities are gathered in the purple cluster. The turquoise cluster introduces the phrase “accessible information” alongside “usability” in relation to Easy-to-Read texts for e-government and public services websites as well as for the internet in general, while the orange cluster presents research carried out in natural language processing for cognitive accessibility purposes, i.e., interfaces and web systems. Lexical simplification to facilitate text processing for people with dyslexia is the area of research in the brown cluster, while, in the pink cluster, the link between accessibility and health information is again highlighted.

## 5. Conclusions

The United Nations (UN) Convention on the Rights of Persons with Disabilities (UNCRPD) [1] condemns any form of “discrimination on the basis of disability” as it is inhumane, unjust, and unequal. The best way to stop discrimination against people with disabilities is to ensure accessibility. Recently, there have been numerous accessibility initiatives for people with disabilities, equal access to services, and products for all members of society in terms of information and communication, made possible through simplified, accessible language, i.e., Easy-to-Read. Easy-to-Read is a concept with vast implications for society, and researching and developing it will prove useful for all of humanity.

This paper’s purpose was to conduct extensive research on the scientific literature from a chronological (publications from 1978 to 2021) and content point of view in order to grasp the evolution of the term as well as its uses or misuses in different fields by the researchers, being vital for an “X-ray” on the current status and in shaping the future trends in this field of research.

The results of the analysis highlight the fact that there is a limited number of publications that include the Easy-to-Read term, that interest in this topic is recent, and that the number of authors involved in such studies is also very low. Limited interest in this area is of concern when attention to this topic may result in equal opportunities, a reduction in discrimination, as well as the development of a sustainable society [53,54,55,56]. Consequently, we call on universities and stakeholders to act as catalysts for social changes by making Easy-to-Read a focus of their agenda.

The keyword analysis that was performed is indicative of the researchers’ attention (*Easy-to-Read* and *intellectual disabilities* representing major concerns in many publications around which the research was centered); future trends regarding the topics to be further investigated can be thus outlined. 

It should be noted that, to date, no bibliometric analysis or systematic review on the literature has been conducted regarding Easy-to-Read and its importance in shaping the future of the accessible documents. Chinn and Homeyard’s meta-narrative literature review on the use of the term has only taken into account the health information publications found mainly in the health databases and focused on materials written for people with intellectual disabilities [16]. 

This analysis provides an overview of the scientific publications concentrating on the use of the Easy-to-Read concept and on the information that can help to improve research strategies in this field by identifying the topics under investigation in this respect. Therefore, it could be used as a good starting point for researchers who seek to identify those areas where such scientific publications are missing. However, it must be mentioned that this study has limitations. The main limitation concerns the fact that the information was collected from a single database, i.e., Web of Science. Although Web of Science contains many publications, there is a definite need to expand the research by including other databases (e.g., Scopus), thus paving new paths for future researchers in this field.

This study’s findings can also help policymakers to plan a national strategy for the creation of Easy-to-Read documents or for making websites more accessible. At the organizational level, the use of Easy-to-Read documents can lead to an improvement in the organizational culture by promoting accessibility and inclusion. At the same time, it can also help to improve communication, with the information being expressed in a clear and easy-to-understand manner. Thus, better and faster decisions can be made, and, as a result, work productivity can be increased; moreover, conflicts arising from the misunderstanding or misinterpretation of the information can be reduced, and ultimately, the organization’s overall efficiency can be improved.

## Figures and Tables

**Table 1:** The most cited publications in the database.

Authors	Title	Research Areas	Times Cited, Web of Science Core	Year
Mayeaux, EJ; Murphy, PW; Arnold, C; Davis, TC; Jackson, RH; Sentell, T	Improving patient education for patients with low literacy skills	General & Internal Medicine	143	1996
Plimpton, S; Root, J	Materials and strategies that work in low-literacy health communication	Public, Environmental & Occupational Health	73	1994
Wilson, FL; Racine, E; Tekieli, V; Williams, B	Literacy, readability, and cultural barriers: critical factors to consider when educating older African Americans about anticoagulation therapy	Nursing	71	2003
Davis, TC; Berkel, HJ; Arnold, CL; Nandy, I; Jackson, RH; Murphy, PW	Intervention to increase mammography utilization in a public hospital	Health Care Sciences & Services; General & Internal Medicine	60	1998
Karreman, J; van der Geest, T; Buursink, E	Accessible website content guidelines for users with intellectual disabilities	Psychology; Rehabilitation	38	2007
Fajardo, I; Avila, V; Ferrer, A; Tavares, G; Gomez, M; Hernandez, A	Easy-to-read Texts for Students with Intellectual Disability: Linguistic Factors Affecting Comprehension	Psychology; Rehabilitation	37	2014
Dowse, R; Ramela, T; Browne, SH	An illustrated leaflet containing antiretroviral information targeted for low-literate readers: Development and evaluation	Public, Environmental & Occupational Health; Social Sciences-Other Topics	35	2011
Chinn, D; Homeyard, C	Easy read and accessible information for people with intellectual disabilities: Is it worth it? A meta-narrative literature review	Health Care Sciences & Services; Public, Environmental & Occupational Health	33	2017
Caposecco, A; Hickson, L; Meyer, C	Assembly and Insertion of a Self-Fitting Hearing Aid: Design of Effective Instruction Materials		25	2011
Sutherland, RJ; Isherwood, T	The Evidence for Easy-Read for People with Intellectual Disabilities: A Systematic Literature Review	Health Care Sciences & Services; Rehabilitation	25	2016
Hurtado, B; Jones, L; Burniston, F	Is Easy Read information really easier to read?	Education & Educational Research; Genetics & Heredity; Neurosciences & Neurology; Psychiatry; Rehabilitation	23	2014
Yaneva, V; Temnikova, I; Mitkov, R	Accessible Texts for Autism: An Eye-Tracking Study	Computer Science; Engineering	17	2015
Dowe, MC; Lawrence, PA; Carlson, J; Keyserling, TC	Patients’ use of health-teaching materials at three readability levels	Nursing	16	1997
Tsai, JHC	Psychometric evaluation of the Demands of Immigration Scale with Taiwanese-Chinese immigrants: a pilot study	Nursing	16	2002
Mejia, A; Filus, A; Calam, R; Morawska, A; Sanders, MR	Measuring Parenting Practices and Family Functioning with Brief and Simple Instruments: Validation of the Spanish Version of the PAFAS	Psychology; Pediatrics; Psychiatry	15	2015
Petterson, T; Dornan, Tl; Albert, T; Lee, P	Are information leaflets given to elderly people with diabetes easy to read	Endocrinology & Metabolism	14	1994
Turnpenny, A; Caiels, J; Whelton, B; Richardson, L; Beadle-Brown, J; Crowther, T; Forder, J; Apps, J; Rand, S	Developing an Easy Read Version of the Adult Social Care Outcomes Toolkit (ASCOT)	Psychology; Rehabilitation	13	2018

## Data Availability

Not applicable.
